# Origin of sexual dimorphism in osteoarthritis risk: the impact of pregnancy and parental care

**DOI:** 10.1186/s12889-026-27899-9

**Published:** 2026-06-02

**Authors:** Supratim Das, Mahdie Rafiei, Marieca-Joelina Burghardt, Jan Baumbach, Linda Baumbach

**Affiliations:** 1 Institute for Computational Systems Biology, University of Hamburg, Hamburg, Germany; 2 Department of Health Economics and Health Services Research, University Medical Center Hamburg-Eppendorf, Hamburg, Germany; 3 Department of Mathematics and Computer Science, Computational Biomedicine Lab, University of Southern Denmark, Odense, Denmark; 4 Centre for Bioinformatics, University of Hamburg, Hamburg, Germany

**Keywords:** Sexual dimorphism, Ethology, Behavioural Biology, Osteoarthritis, Epidemiology, Pregnancy Hormones

## Abstract

**Background:**

A higher osteoarthritis (OA) risk in females is well-documented, but the causes of this sexual dimorphism remain unclear. This study explores whether this disparity arises from sex differences in development or the sex difference in total reproductive costs of gestation and parental care.

**Method:**

We tested four hypotheses on the effects of gestation and parental care on OA risk using data from the German Aging Survey (DEAS), controlling for age, education, and comorbidities, as appropriate. We analyzed 2,328 men and 2,359 women with children, and 313 men and 319 women without children. We compared OA risk (1) between childless men and women, (2 and 3) within each sex based on parental status, and (4) by the number of children, stratified by sex. We used logistic regression for each test and calculated adjusted 95% confidence intervals and p-values. First test the effect of sex difference in risk development, while the second and third determine the effect of total reproductive costs in each sex, and the fourth examines the dose-dependent effect of reproductive cost in OA risk development.

**Results:**

No significant difference in OA risk was found between childless men and women (37% and 44%, respectively, CI = -0.035 to 0.602, *p = *0.081). However, women with children had a significantly higher OA risk than childless women (51%, and 44%, respectively, CI = 0.004 to 0.483, *p = *0.047). Differences in risk between men with and without children were insignificant ( 43%, and 37%, respectively, CI = -0.069 to 0.432, *p = *0.156). OA risk increased significantly with the number of children in women (0.10, CI = 0.03 to 0.179, *p = *0.006,) but not in men (0.02, CI = -0.058 to 0.091, *p = *0.660,) as the previous experiment also suggests.

**Conclusion:**

Sexual dimorphism in OA risk appears to be driven by the reproductive costs of gestation and parental care. Our findings highlight the need for finer stratification based on reproductive status to better understand sex differences in disease risks.

**Supplementary Information:**

The online version contains supplementary material available at 10.1186/s12889-026-27899-9.

## Abstract (plain language summary)

Osteoarthritis is more common in women than in men. Scientists have long known about this difference, but the reasons behind it are unclear. This study looked at whether the higher risk of osteoarthritis in women is linked to biological differences between the sexes or to pregnancy and parenting.

We analyzed data from thousands of men and women in Germany, comparing those with children to those without. We found that childless men and women had similar rates of OA, suggesting that biological sex alone may not explain the difference. However, women with children were more likely to develop osteoarthritis than childless women. The risk of osteoarthritis also increased for women as the number of children they had grew. For men, having children did not seem to affect their osteoarthritis risk.

These findings suggest that pregnancy and parenting may play a key role in why women are more likely to develop osteoarthritis. This study highlights the importance of considering reproductive history when studying health differences between men and women. By understanding these factors better, doctors and researchers can work toward improving prevention and treatment for osteoarthritis in men and women.

**Supplementary Information:**

The online version contains supplementary material available at 10.1186/s12889-026-27899-9.

## Highlights

Women have a higher risk of osteoarthritis (OA) compared to men, but this study found no significant difference in OA risk between childless men and women.

Women with children have a significantly higher OA risk than childless women, while parental status does not affect OA risk in men.

The number of children is directly associated with increased OA risk in women, but not in men.

The sex difference in OA risk appears to be driven by the differential reproductive costs of pregnancy and parental care, rather than differences in biological development between sexes.

These findings emphasize the importance of considering reproductive history to better understand sex differences in disease risks.

**Supplementary Information:**

The online version contains supplementary material available at 10.1186/s12889-026-27899-9.

## Introduction

### Background

Osteoarthritis(OA) affects 1 in 5 US adults [1] and is a debilitating joint disease characterized by changes in cartilage, the occurrence of pain, stiffness, and reduced mobility [2]. It is a major cause of disability worldwide, affecting millions of individuals and imposing a significant burden (a total of 136.8 billion USD in the US alone [3]) on the healthcare system [4]. Notably, epidemiological studies have consistently shown that females are at a higher risk of developing osteoarthritis compared to males [5–9, 9–13]. This sexual dimorphism in osteoarthritis risk has been recognized both epidemiologically and physiologically [14–17], yet the underlying mechanisms remain poorly understood.

The increased risk of osteoarthritis in females can be attributed to a combination of biological, hormonal, and socio-cultural factors [18]. However, the relative contributions of these factors are not well delineated. One area of interest is the role of pregnancy and the associated endocrinological transitions. Pregnancy induces significant hormonal changes, including elevated levels of estrogen, progesterone, and relaxin, which can impact joint health [19–21]. Estrogen and progesterone influence the production of collagen and other components of the joint matrix. In female rodents, sex hormones, particularly estradiol, and androgens protect against inflammation-induced cartilage degradation [22]. Thus, these hormonal fluctuations could also lead to joint instability in women, potentially contributing to the development of osteoarthritis.

Weight gain during pregnancy is another critical factor. The additional weight places acute increased stress on weight-bearing joints such as the knees and hips [23], accelerating cartilage degeneration [24] since the muscles can not adjust quickly enough. Furthermore, the biomechanical changes associated with pregnancy, including altered gait and posture, can lead to abnormal joint loading and increased risk of joint damage [25]. These physiological and biomechanical changes could contribute to the complex interplay between pregnancy and osteoarthritis risk.

Parental care can increase osteoarthritis risk due to both biological and socio-cultural factors [26]. Physical activities like lifting, carrying, bending, and kneeling strain the joints and accelerate cartilage wear [27]. Stress from parental responsibilities raises cortisol levels [28], contributing to inflammation and joint damage [29]. Sleep deprivation impairs joint tissue repair as shown in rodent model organisms [30, 31]. Sleep deprivation is also shown to exacerbate pain, fatigue, and rheumatoid arthritis-related joint pain in humans [32]. Additionally, parents often have less time for regenerative exercise [33], which is recommended as prevention for osteoarthritis [34, 35]. It is also shown that marriage, parenthood, and employment adversely affect men’s time for exercise more than that of women [33].

To understand the origin of sex differences in osteoarthritis risk, it is crucial to disentangle the effects of the reproductive cost of gestation and parental care from developmental sex differences. In evolutionary biology, reproductive cost refers to the trade-off where investing energy, resources, or time in producing and raising offspring reduces an organism’s future survival, growth, or additional reproduction. This stems from limited resources, forcing allocation choices between current reproduction and somatic maintenance or future breeding bouts. Parental care heightens reproductive costs by diverting parental resources to offspring survival, such as protection or feeding, which boosts offspring fitness but often lowers parental longevity or additional reproduction chances. The reproductive cost is different for different sex often causing differential selection pressure leading to sexual dimorphism. So far, the reasons for sex differences in osteoarthritis have been investigated in only one study, which is limited to a Danish cohort [26]. This geographically limited cohort from Denmark suggests an increase in osteoarthritis risk in both sexes due to parental care [26]. In the study, the authors didn’t focus on disentangling factors for the origin of sex differences. They found short education, low income, and married status to be significantly associated with increased OA risk, and persons with children were at higher risk in both men and women. However, their study included data from the years between 1982 and 2008. The cultural component of parental care might have changed until today [36], as the roles of mother and father have changed over the years. Hence, here we use data from a bigger geographical area and include participants from more recent years to validate the findings.

From a clinical standpoint, in osteoarthritis, sex (male vs female) has been used as a risk factor [12]. Further understanding the mechanisms underlying the increased osteoarthritis risk in females is crucial for developing effective prevention and treatment strategies. In the current paradigm, sex is thought of as a risk factor in osteoarthritis. We aim to find if sex in itself is a risk factor for osteoarthritis or if it’s the gestation, parental care, and/or the number of children.

### Objective

**Fig. 1:**
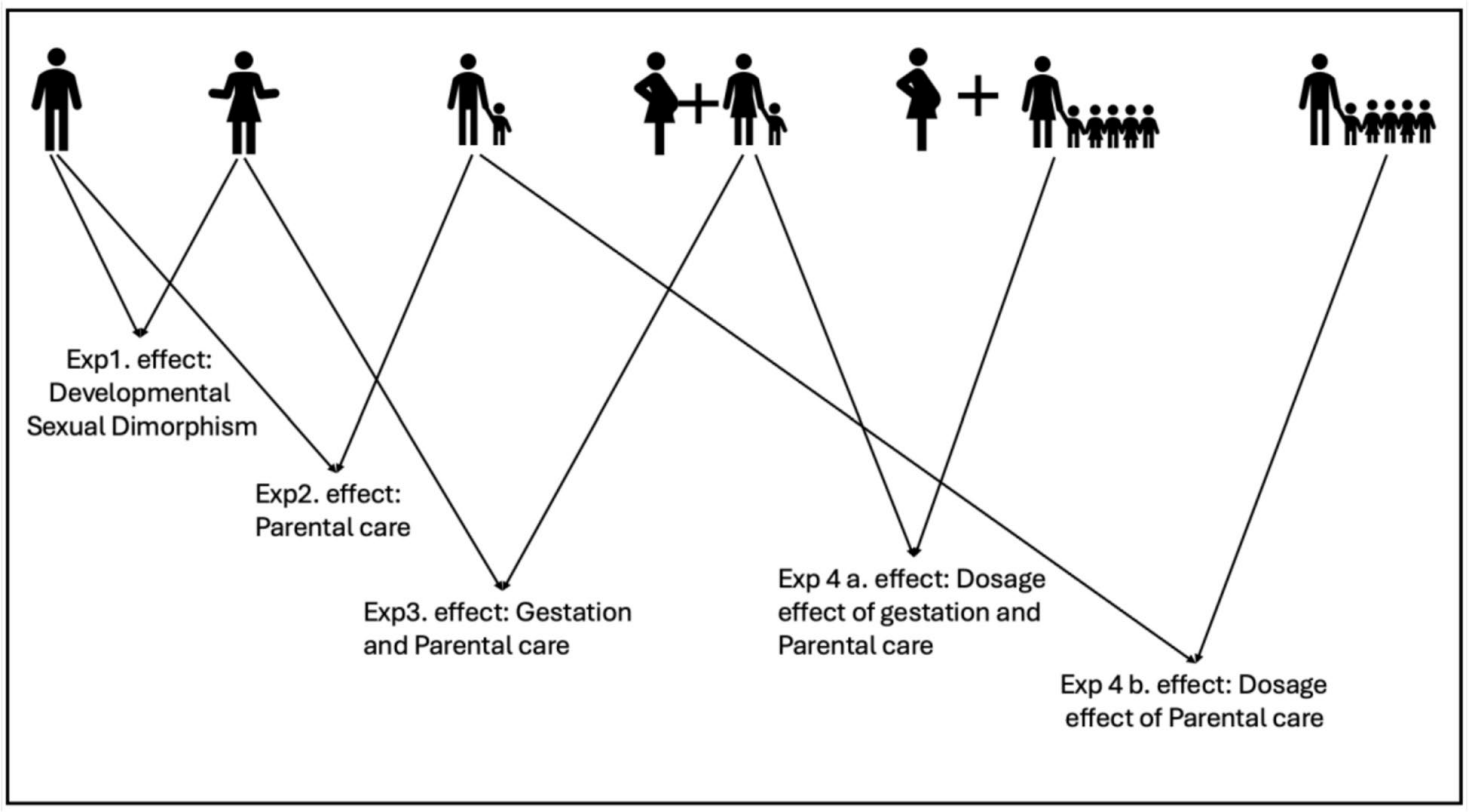
Origin of sexual dimorphism in osteoarthritis: Representation of different groups used for stratification and comparison of osteoarthritis risk. The icons indicate the groups considered in our experiments, and the arrows indicate the groups compared in each experiment. The text indicates the factors that were tested through that experiment. For example, in Experiment 1 (Exp1) we check if there is any difference in the risk of osteoarthritis in men and women without children. If we find any difference, we will conclude that this arises because the developmental sexual dimorphism affects osteoarthritis risk in the two sexes differently. Similarly, in experiment 2 by comparing men with and without children, we evaluate the effect of men’s parental care. In experiment 3, we evaluate the effect of gestation and women's parental care. In Experiment 4, we evaluate if there is a dosage effect with the number of children in either sex. Exp: Experiment

In our studies, we want to disentangle the effects of gestation and parental care from developmental sex differences in osteoarthritis risk. We hypothesize that the sexual dimorphism in osteoarthritis is not a mere developmental sex difference; rather, pregnancy and the differential cost of parenteral care cause the sexual dimorphism. Our specific hypotheses are the following (see Fig. 1):

1. Sex differences in osteoarthritis risk without children: Null hypothesis: men and women without children exhibit no different osteoarthritis risk burdens. (see Fig. 1. Exp. 1).
2. Osteoarthritis risk in women with and without children: Null hypothesis: women with children have no difference in osteoarthritis risk burden compared to women without children.(see Fig. 1. Exp. 2).
3. Osteoarthritis risk in men with and without children: Null hypothesis: men with children have no difference in osteoarthritis risk burden compared to men without children.(see Fig. 1. Exp. 3).
4. Dose dependence of osteoarthritis on the number of children in men and women: Null hypothesis: The number of children doesn’t influence osteoarthritis risk in either sex in a dose-dependent way. (see Fig. 1. Exp. 4.a and Exp. 4.b).

Further details of confounder selection and the rationale behind the hypothesis are described in detail in the method section under the “ Statistical tests and rationale” subheading.

## Method

### Study design

Our observational study aims to disentangle the effects of developmental sex difference, pregnancy, and parental care on osteoarthritis risk by utilizing data from the German Aging Survey (DEAS) [37]. To address the gap in understanding the relative contributions of these three factors, we formulated four hypotheses. We followed the STROBE guidelines [38] to present our study findings.

### Setting and data source

The German Aging Survey (DEAS) is a nationwide representative survey of the German population aged 40 and older. Included participants must reside in private households in Germany and be between 40 and 85 years of age. The DEAS [37] contains multiple survey waves and was initiated in 1996. Survey waves were conducted in 1996, 2002, 2008, 2011, 2014, 2017, 2020, and 2020/21. We used data from Wave 2017 as it includes our desired variables and isn’t affected by the COVID-19 pandemic. The DEAS uses a multi-stage random sampling method to ensure a representative cross-section of the German population. Data is collected through face-to-face interviews and self-administered questionnaires. The data collected covers various aspects of life, including health, economic situation, social participation, family and social networks, and subjective well-being. The data collection for the DEAS is managed by the German Centre of Gerontology (DZA). The DZA is responsible for conducting the survey, processing the data, and making it available for scientific research; this data is open source and can be requested through the DZA’s website.

### Participants and variables

We included participants providing complete information on the following variables: osteoarthritis, age, sex, number of children, level of education, BMI and 17 binary variables on the presence of certain diseases as described in detail below. We excluded the participants who had missing values for at least one of the selected variables. Our independent variable of interest was OA risk. Our main independent variables were sex and having children; the remaining variables were considered as potential confounders.

For osteoarthritis, we used the following variable: “Suffering from illnesses: Joint degeneration (arthrosis) of the hips, knees, or spine”. It’s important to note that the actual question asked is also used to obtain information on additional diseases. It is introduced by: “​​I will now read you a list of diseases. Please tell me whether or not a doctor has told you that you are suffering from any of the following diseases.” The questionnaire differentiates osteoarthritis from rheumatoid arthritis and osteoporosis and includes a total of 20 diseases.

The level of education was obtained with a 4-category variable—low (without A level or vocational qualification), medium (with A level or vocational qualification), sophisticated (master craftsmen, technical school, or similar), and high (Respondents with university degrees).

The question to evaluate the number of children was: “Do you have children? By this I mean children of your own, children who have grown up or are growing up in your household, as well as any children who may no longer be alive.” For our analyses, we used a dichotomous variable of having children vs. not having children, but for our last hypothesis, we kept the number of the indicated children up to 3. However, we merged four or more children into one category due to a low number of data points.

The variables on suffering from 17 diseases (out of a total of 20, including osteoarthritis, osteoporosis, and rheumatoid arthritis) were also used to create one single variable called comorbidity, which included the number of all diseases. The list of diseases was as follows: 1. Increased blood fat levels and cholesterol levels; 2. Diabetes, increased blood sugar levels; 3. High blood pressure, 4. Heart attack, angina pectoris; 5. Cardiac insufficiency, including coronary artery disease; 6. Stroke, 7. Circulatory disorders of the brain; 8. Circulatory disorders of the legs; 9. Chronic lung disease (e.g., chronic bronchitis, pulmonary emphysema); 10. Cancer, malignant tumor (including leukemia); 11. Gastric ulcer, duodenal ulcer; 12. Incontinence, 13. Mental illness, 14. Parkinson's disease; 15. Glaucoma, 16. Other chronic diseases; 17. Other diseases. While calculating the comorbidity score, we didn’t use the following three diseases as they are related to osteoarthritis: 1. Osteoporosis, 2. Joint degeneration (arthrosis) of the hips, knees, or spine, 3. Inflammatory joint or spinal disease (arthritis or rheumatoid arthritis). Thus, the maximal number of comorbidities, in theory, can be 17, while the minimum can be 0. The prevalence of these diseases across different groups (childless men, men with children, childless women, women with children) is reported in the supplementary table S1.

### Statistical tests and rationale

To address the impact of various biological and cultural factors on sex differences in OA risk, we conducted four statistical hypothesis tests. For all the tests, we used logistic regression tests and a p-value significance threshold of 0.05. We chose logistic regression as the target variable of interest (i.e., osteoarthritis) was a binary variable. All analyses were performed in Python.

#### Sex differences in osteoarthritis risk without children

We examined whether men and women without children exhibit different osteoarthritis risk burdens. A lack of differential risk or a reduction in the difference compared to the overall male vs. female population would indicate that sex has minimal or no effect on osteoarthritis risk. This finding would suggest that biological differences, potentially due to sex hormones and their influence from fetal development to adulthood, play a role.

For this experiment, we did not include any confounding variables. As sex doesn’t have any causal path from BMI, level of education, comorbidity, or age, in a causal direct acyclic graph (DAG) there are no open biasing paths, so we didn’t need to make any adjustments. We have provided the causal DAG used for selecting or not selecting variables in the supplementary figure 1.A.

#### Osteoarthritis risk in women with and without children

We assessed whether women with children have a different osteoarthritis risk burden compared to women without children. If no differential risk is observed, it would imply that gestation and parental care do not increase osteoarthritis risk. Conversely, a significant differential risk would indicate an effect of these factors on osteoarthritis risk.

For this experiment, we used BMI and level of education as confounders. The rationale behind it is that a higher level of education is usually achieved before a person reaches this age, and it could have affected the number of children. Similarly, pregnancy-modulated gain in BMI persists even 40 years later [39], and BMI is a well-known risk factor for OA [40]. However, we didn’t add comorbidity here as a confounder for adjustment, as BMI affects comorbidity, thus its inclusion would have caused overcorrection. As our dataset considers an aging population, the effect of age on the number of kids for women would be very limited, hence we didn’t use age as a confounder here. We have provided the causal DAG used for selecting or not selecting variables in the supplementary figure 1.B.

#### Osteoarthritis risk in men with and without children

We investigated whether having children alters osteoarthritis risk in men by comparing men with and without children.

For this experiment, we used age, education, and BMI as confounders. For men As comorbidity is correlated with BMI, to avoid overcorrection, we didn’t add comorbidity as a confounder here. Unlike women, for men, age affects the number of kids as they have longer reproductive periods. We added education and comorbidity as it affect the number of kids as well as OA for men. We have provided the causal DAG used for selecting or not selecting variables in the supplementary figure 1.C.

#### Dose dependence of osteoarthritis on the number of children in men and women

Additionally, we explored whether the number of children influences osteoarthritis risk in both sexes. We grouped having children into having 0, 1, 2, 3, or 4 or more children due to limited sample sizes for individuals with more than four children. We performed logistic regression with the number of children as the independent variable and osteoarthritis risk as the target variable. A significant positive slope would indicate a dose-dependent relationship between the number of children and osteoarthritis risk, whereas a slope close to zero with an insignificant *p*-value would suggest no such relationship. For this experiment, for women, BMI and education were used as confounders, while for men, education, age, and BMI were used as confounders for the same reason as explained in experiments 2 and 3 (in sections 2.4.2 and 2.4.3, respectively).

## Results

### Participants and variables

After dropping cases with missing values (83 participants, i.e., 1.5% of the sample size), we could include 5,319 participants. Out of which 2,678 were females, and 2,641 were males. The following Table 1 presents the characteristics of the included participants stratified by sex and parenthood.

**Table 1:** Characteristics of the included participants stratified by sex and parenthood

Characteristic	Men without children	Women without children	Men with children	Women with children
Number of Participants	313	319	2,328	2,359
Number of Participants with Osteoarthritis	116 (37%)	140 (44%)	1005 (43%)	1200 (51%)
Age	Mean: 65.67	Mean: 66.52	Mean:68.25	Mean:69.65
	SD: 10.27	SD: 9.87	SD: 10.25	SD: 10.52
Level of Education				
1: low	1: 14 (4.39%)	1: 4 (1.28%)	1: 174 (7.38%)	1: 35 (1.5%)
2: medium	2: 150 (47.02%)	2: 142 (45.37%)	2: 1,262 (53.5%)	2: 949 (40.76%)
3: sophisticated	3: 46 (14.42%)	3: 49 (15.65%)	3: 326 (13.82%)	3: 366 (15.72%)
4: high	4: 109 (34.17%)	4: 118 (37.7%)	4: 597 (25.31%)	4: 978 (42.01%)
Comorbidity 0–17)	Mean: 2.22	Mean: 2.23	Mean: 2.24	Mean: 2.30
	SD: 1.52	SD: 1.57	SD: 1.71	SD: 1.65
BMI	Mean: 27.39	Mean:25.76	Mean: 27.12	Mean: 26.24
	SD: 5.07	SD: 5.39	SD: 3.92	SD: 4.90

### Statistical tests

#### Sex differences in osteoarthritis risk without children

**Fig. 2:**
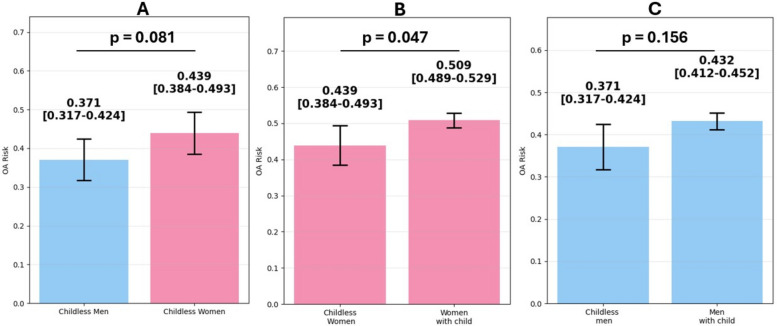
Results of the first three hypothesis testing: (**A**). Men without children vs. women without children, (**B**). Women with vs without children (**C**). Men with vs. without children. Y axis is OA risk. Error bars depict the adjusted 95%CI, the numbers above each column represent the mean value along with 95% CI margins in brackets

**Fig. 3:**
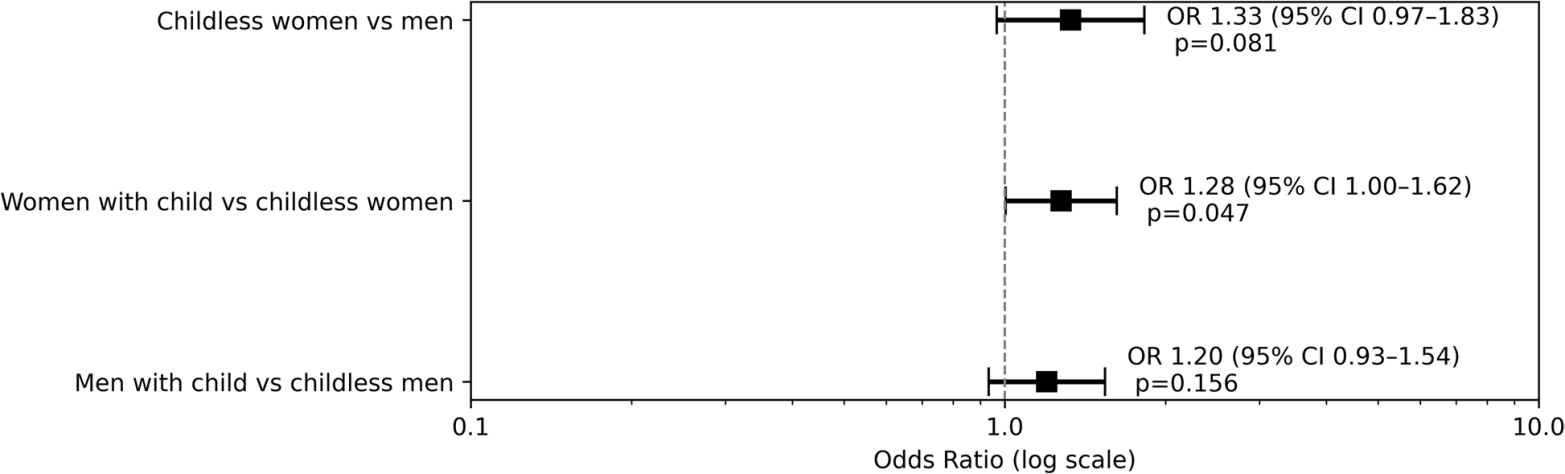
Forest plot of the first three hypothesis testing: From top to bottom 1. Men without children vs. women without children, 2. Women with vs without children 3. Men with vs. without children. The X-axis is the odds ratio on a log scale, and the error bars represent the 95% CI of the effect size (odds ratio)

Men without children have a 37% risk of having OA while women without children have a 44% risk of having OA (See Fig. 2.A. and Fig. 3). For men without children and women without children, we found no statistically significant difference (*p*-value = 0.081, see Table 2) in osteoarthritis risk burden.

**Table 2:** Results of hypothesis testing

Experiment	Confounding variable for adjustment	Coefficient	95%CI	*P*-value
1. OA risk difference in childless men against childless women	none	0.284	−0.035 to 0.602	0.081
2. OA risk difference in women without children against women with children	BMI, level of education	0.243	0.004 to 0.483	0.047
3. OA risk difference in men without children against men with children	BMI, level of education, age	0.182	−0.069 to 0.432	0.156
4. Dose-dependent effect of the number of children on OA in women	BMI, level of education	0.105	0.03 to 0.179	0.006
5. Dose-dependent effect of the number of children on OA in men	BMI, level of education, age	0.017	−0.058 to 0.091	0.660

#### Osteoarthritis risk in women with and without children

Women with children have a 51% risk of having OA while women without children have a 44% risk of having OA. We found a statistically significant difference (p-value = 0.046, see Table 2) in osteoarthritis risk burden while controlling for BMI and education level as a confounder. (See Fig. 2. B. and Fig. 3).

#### Osteoarthritis risk in men with and without children

Men with children have a 43% risk of having OA, while men without children have a 37% risk of having OA. We found no statistically significant difference (*p = *0.131, see Table 2) in osteoarthritis risk burden while controlling for education level, age, and comorbidities as confounders. [See Fig. 2.C. and Fig. 3].

#### Dose dependence of osteoarthritis on the number of children in men and women

**Fig. 4:**
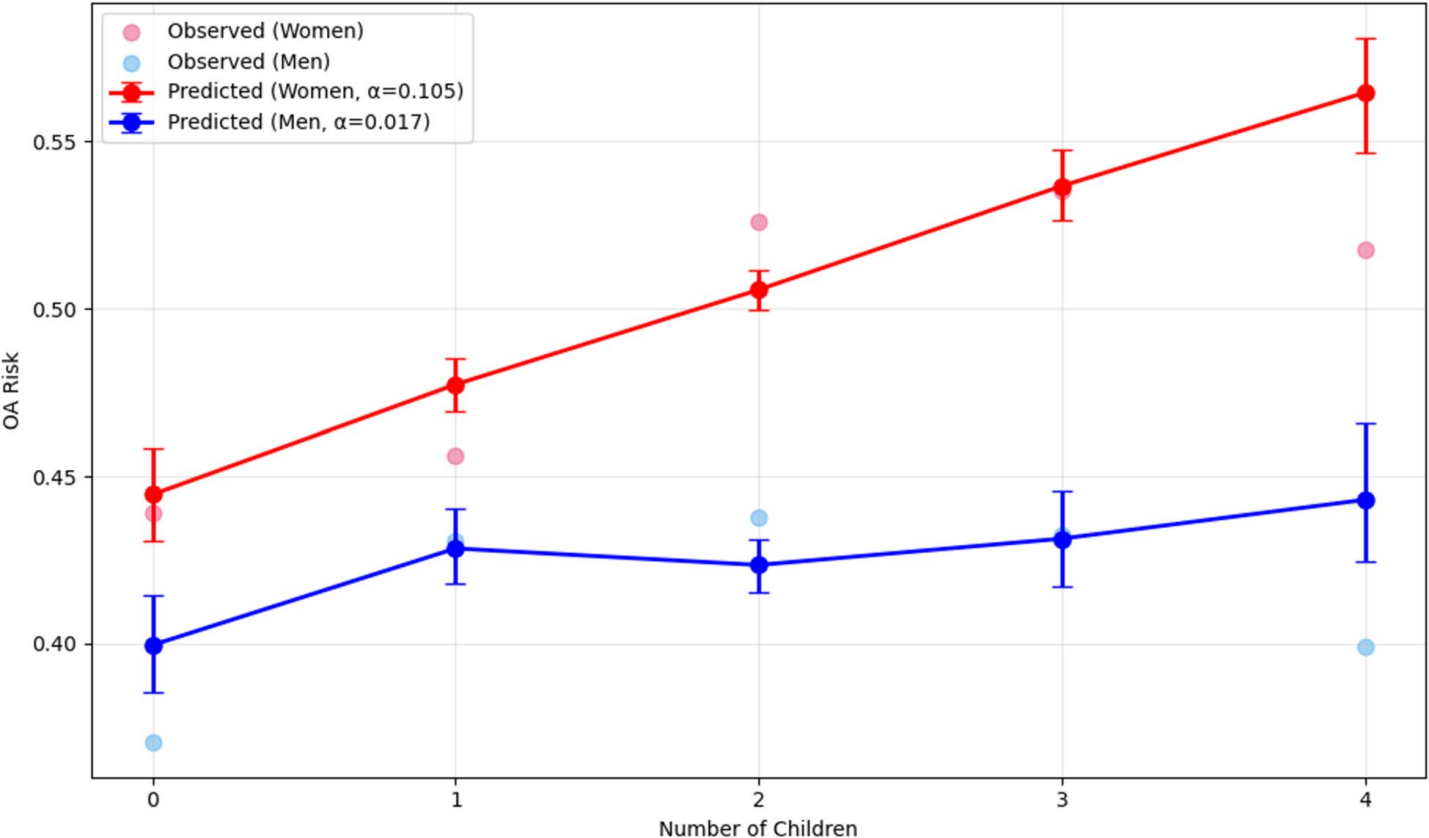
Risk of osteoarthritis with an increasing number of children: The red line on top represents women, and the blue line underneath represents men. The error bars represent adjusted 95%CI

**Fig. 5:**
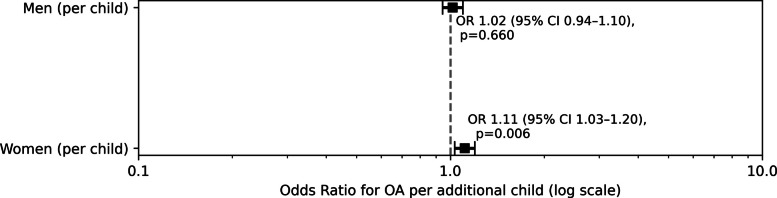
Forest plot of osteoarthritis with an increasing number of children: X-axis is the odds ratio (in log scale) for an increase in OA risk per additional child, and the error bars represent the 95% CI of the effect size (odds ratio)

We found a positive slope of 0.12 with a p-value of 0.013 for the trendline for females (controlling for bmi and education level as a confounder), while for males, the slope (0.02) is close to zero (controlling for education level, age, and comorbidities as confounders), and the p-value is insignificant (0.660). (See Fig. 4. and Fig. 5). For this analysis, our cohort contained 319 women without children, 618 women with one child, 1158 women with two children, 413 women with three children, and 170 women with four or more children. Similarly, we had 313 men without children, 583 men with one child, 1124 men with two children, 423 men with three children, and 198 men with four or more children.

## Discussion

From the statistical tests, we find that men and women without children have no statistically significant difference in the risk of OA. Amongst women, participants with children have a statistically significantly higher risk of developing osteoarthritis than those without. Among men, participants with or without children have no statistically significant difference in osteoarthritis risk. We also see that, in women with an increasing number of children, the risk increases. In men, we found that men with children do not exhibit a statistically significant difference in osteoarthritis risk compared to men without children, nor does the risk increase with an increasing number of children. Note that we didn’t perform any Bonferroni correction as we only performed five tests.

Based on the results of the above tests conducted on participants of the German Aging Survey (DEAS), we can infer that the sex differences in total reproductive cost due to gestation and the differential cost of parental care are contributors to sexual dimorphism in osteoarthritis rather than developmental sex differences alone. Consequently, gestation should be considered a risk factor in the assessment of osteoarthritis risk. Our analysis indicates that, although men with children on average have ~ 5% high OA risk than those without children, it’s not statistically significant; hence, we conclude parental care elevates the risk of osteoarthritis with statistical significance only in women. In females, the number of children has a significant and dose-dependent impact on osteoarthritis risk, leading to sexual dimorphism across the entire population. These findings highlight the necessity of considering both biological and sociocultural factors to fully understand the sexual dimorphism in osteoarthritis risk.

This finding conflicts with that of the Danish study [26], which reports a statistically significant difference in OA risk between men with and without children. However, while not statistically significant in our study, the mean risk of OA in men with children is higher than in those without children, aligning with the findings of the Danish study. It is also noteworthy that the Danish study [26] found an even greater difference in women than in men, indicating the impact of parental care. We hypothesize that the extent of the differential cost of parental care depends on cultural context, which may vary with time and geographical location. Further studies are required to determine if this trend is consistent across populations beyond the German and Danish cohorts. It is also important to note that our study design does not disentangle the effect of gestation and female parental care in females. The cost of parental care itself differs between males and females [41]. Although our study did not find a significant effect of male parental care on OA risk, the Danish study indicates that even male parental care contributes to the OA risk burden. Overall, our study shows that the total reproductive cost of gestation and female parental care has a dose-dependent effect on OA risk burden.

From an evolutionary biology perspective, we also see a dose-dependent response in OA risk for women, unlike men. This may suggest that with each pregnancy, women accumulate a biological burden towards osteoarthritis risk. However, as osteoarthritis is manifested more severely at senescence, this doesn’t affect women’s reproductive fitness, as this occurs after menopause. Hence, we hypothesize that there is not sufficient selection pressure to eradicate the phenotypes causing OA in women, causing the accumulation of this disease in women more than in men.

In the field of biology of sexual diamorphism, non-tournament primate species (i.e., reproductive success spreads more evenly across males due to relaxed dominance hierarchies), it is intriguing to observe sex differences in specific behavioral or physiological traits within populations through observational studies. It is crucial to stratify the population into distinct groups based on relevant contexts, such as the number of children, as this can influence the observed extent of sex differences within certain strata. This approach aids in elucidating the mechanistic origins of sex differences in specific traits, particularly those that predominantly manifest in the post-reproductive phase and have minimal impact on an individual’s evolutionary fitness. Consequently, our research challenges the current ethology paradigm in understanding sexual dimorphism in primate behavior and disease susceptibility.

From an epidemiological perspective, we find in our dataset that sex itself is not a risk factor; rather, it is the number of children in women. Hence, researchers, epidemiologists, and maybe clinicians should consider investigating the presence of children rather than solely the sex of patients when assessing the OA risk. Recent work on personalized outcome predictions for knee OA in structured exercise programs further supports the need for tailored risk assessments to improve treatment strategies [42].

### Limitations

Firstly, for the variable of children, we assumed a linear relationship between the number of children and the number of pregnancies. However, this assumption may not account for cases of adoption and step-children. Our assumption also doesn’t take into account miscarriage or willful termination of pregnancy, although in such scenarios, endocrinological changes could have affected OA risk. Secondly, for the disease variables, we assumed that diagnosed diseases accurately represent the existence of all diseases. However, there may be undiagnosed diseases that are not captured in this data. Thirdly, the variable used for retrieving data on OA is questionnaire-based. Patients thus have to recall the information, which may introduce a bias. Moreover, the variable doesn’t distinguish the difference between the loci of the pain; this information would have been informative, but wasn’t included in the data. For instance, one can expect a higher burden on the knee for pregnancy-related weight gain compared to hand OA. Further studies are required to stratify the reproductive cost burden in each locus of OA. Similarly, DEAS only includes variables on trauma or falls from the last year, which do not affect the reproductive fitness of the present aging population; thus, it was not used as a confounder. However, a history of past trauma could serve as a useful confounder in future studies involving different cohorts. Fourthly, we also need to note that our data cohort represents only data from Germany; further studies are required for the generalization of our conclusion to the global population. Lastly, it’s important to note that although we include only data from the aging population and the results suggest that the reproductive cost of gestation and parental care affects OA risk, our study is cross-sectional, hence we can not confirm causation.

#### Further studies

It’s important to verify if our observed trends hold true in other countries and if the dose-dependent effect of pregnancy on women’s OA risk is caused by differential parental care or differential biological effects. Further studies are required to see if the extent of egalitarianism in society in different countries changes in the rate of accumulation of OA risk burden.

## Conclusion

In conclusion, our study disentangles the effect of sex difference in development itself from the total reproductive costs of gestation and parental care, and osteoarthritis risk. Our work on German data suggests that the origin of sexual dimorphism lies in the difference in total reproductive costs of pregnancy and parental care. We also find that for women, the osteoarthritis risk is dose-dependent on the number of children. Henceforth, further studies are required to reexamine and maybe reformulate the use of sex as a risk factor for osteoarthritis.

## Supplementary Information

Supplementary Material 1

## Data Availability

We used data from the 2017 wave of the German Aging Survey (DEAS) [37], which is open-source data that can be received by submitting an online application and signing a physical copy of the contract. All codes for data preprocessing and analysis can be accessed through the GitHub repository using the [43]. 37. Claudia, V. et al. Scientific Use Files German Ageing Survey (SUFs DEAS) 1996–2021, Version 1.0Scientific Use Files Deutscher Alterssurvey (SUFs DEAS) 1996–2021, Version 1.0. Deutsches Zentrum für Altersfragen https://doi.org/10.5156/DEAS.1996-2021.M.001 (2022). 43.Rafiei, M. https://github.com/Mahdieh-Rafiei/PregOA.

## Abbreviations

OA: Osteoarthritis
DEAS: German Aging Survey
DZA: German Centre of Gerontology
BMI: Body Mass Index
