# Supplementary material for: Origin of sexual dimorphism in osteoarthritis risk: the impact of pregnancy and parental care
Source: BMC Public Health. 2026 Jun 2;26:1866. doi: 10.1186/s12889-026-27899-9 (PMC13270846; doi:10.1186/s12889-026-27899-9)
Supplement: Supplementary file 1 — Supplementary Material 1 [file 12889_2026_27899_MOESM1_ESM.docx]

**Origin of sexual dimorphism in osteoarthritis risk - : The Impact of Childbearing and Parental Care.**

**Supratim Das†*^a,b^, Mahdie Rafiei*^a^, Marieca-Joelina Burghardt^a^, Jan Baumbach^a,c^, Linda Baumbach^b,d^**

Supplementary Material:

Supplementary figure 1:

Method: Causal Direct Acyclic Graphs (DAGs) for each experiment are presented below. These causal DAGs were generated in <https://www.dagitty.net/dags.html>. The webinterface provides information on which variable (represented as a node) needs to be taken into account for adjustment by painting them in red. The directional arrows depicts causality.


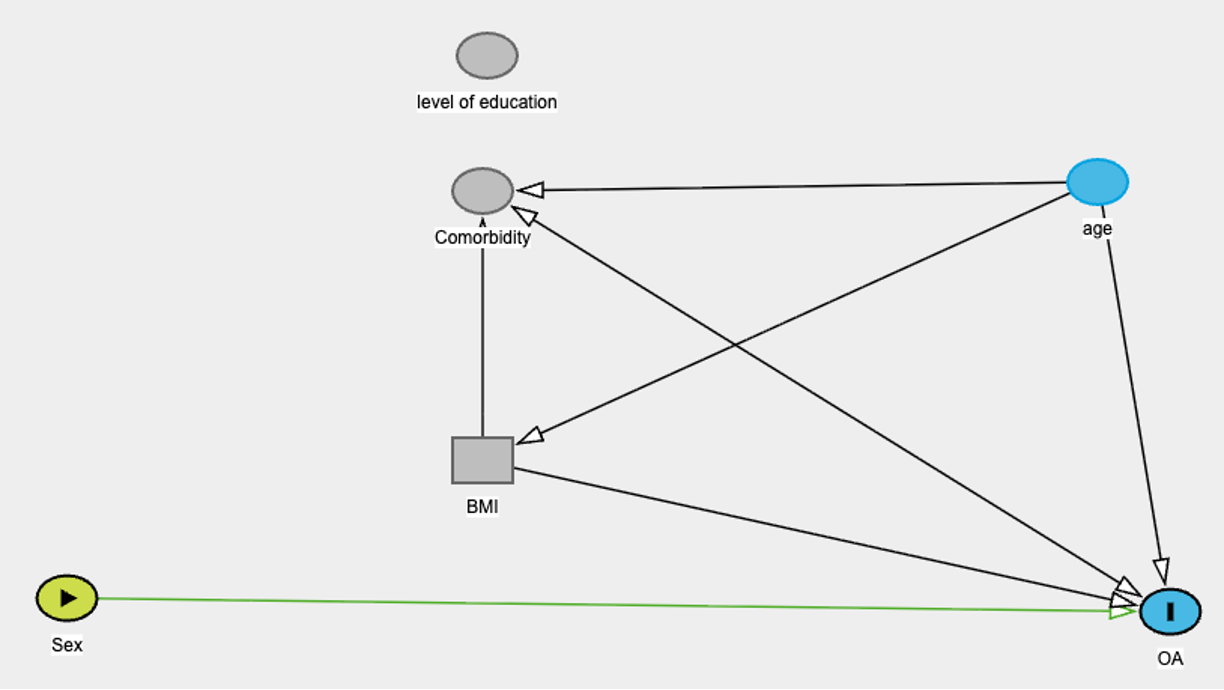


1.A. Experiment 1. OA risk difference in childless men and childless women.


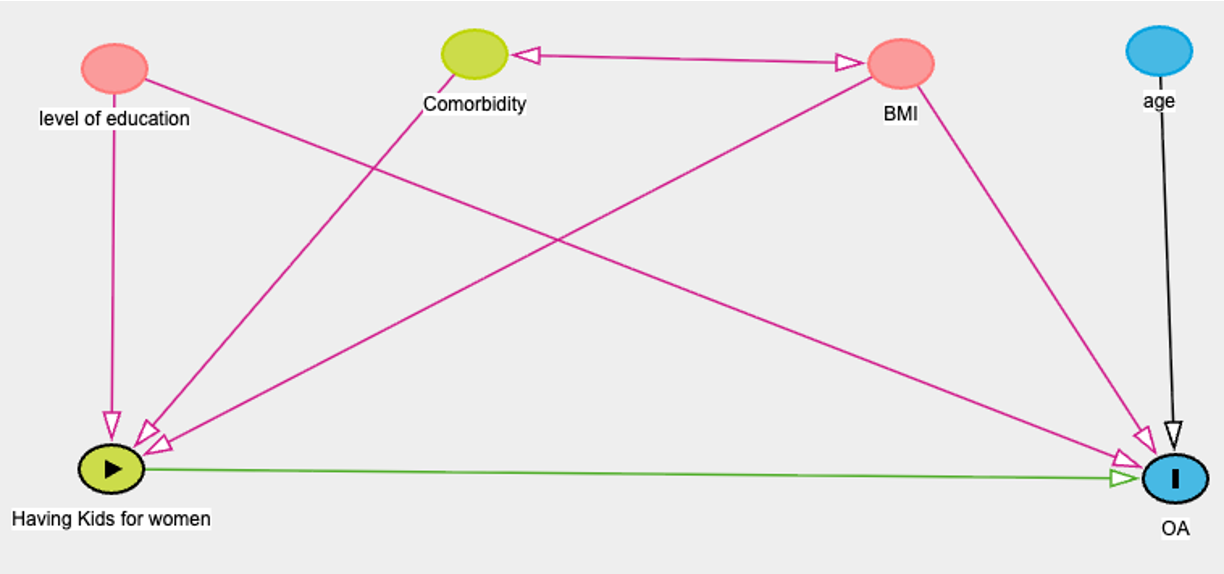


1.B. Experiment 2.OA risk difference in women with and without children


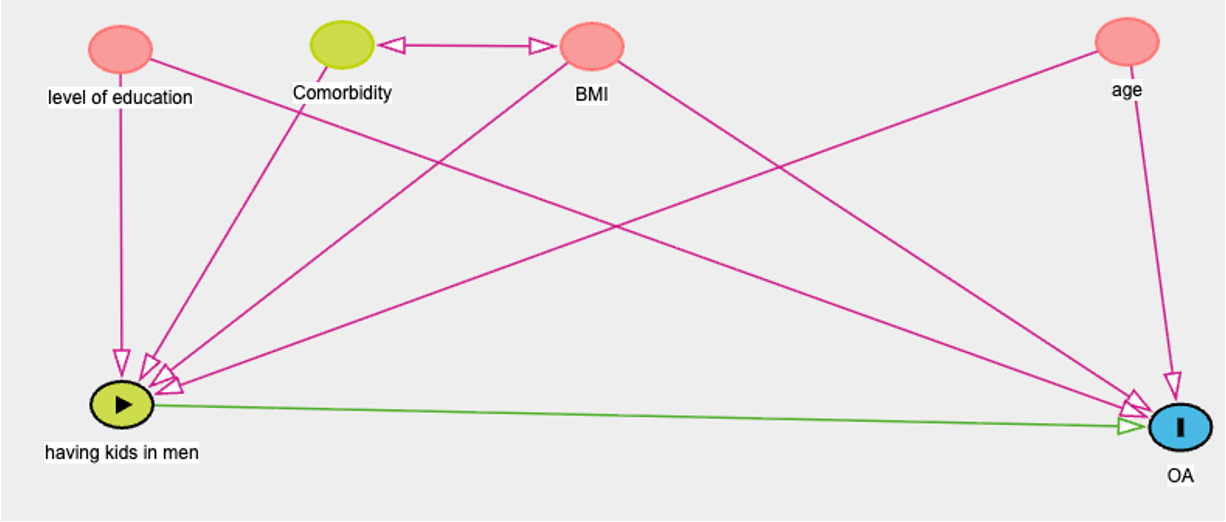


1.C. Experiment 3.OA risk difference in men with and without children

Supplementary Table 1:

Results:

Here we provide a table for the prevalence of all the other diseases which were used to calculate the total comorbidity score.

Table: S1: Prevalence of diseases amongst diffrerent groups.

| Disease | Childless Men | Men with Child | Childless Women | Women with child |
| --- | --- | --- | --- | --- |
| Diabetes | 16.40% | 16.10% | 6.90% | 11.20% |
| High cholesterol | 31.20% | 34.70% | 29.80% | 31.20% |
| blood_pressure | 50.20% | 52.60% | 43.40% | 45.80% |
| Heart_attack | 6.90% | 7.10% | 2.70% | 3.10% |
| Cardiac_insufficiency | 18.30% | 16.00% | 7.50% | 11.40% |
| Stroke | 4.40% | 5.40% | 4.50% | 3.40% |
| Circulatory_disorders_brain | 1.90% | 2.20% | 2.10% | 2.10% |
| Circulatory_disorders_legs | 10.40% | 11.30% | 10.80% | 9.90% |
| Osteoporosis | 3.80% | 4.20% | 12.00% | 13.00% |
| Inflammatory_joint | 10.40% | 14.30% | 19.30% | 16.10% |
| Chronic_pulmonary | 9.20% | 7.70% | 10.50% | 9.30% |
| Cancer | 9.20% | 11.00% | 10.80% | 10.30% |
| Stomach_ulcer | 3.50% | 3.30% | 2.10% | 2.80% |
| Incontinence | 2.20% | 4.50% | 4.50% | 5.30% |
| Mental_illness | 9.80% | 4.70% | 12.60% | 10.90% |
| Parkinson_disease | 1.60% | 1.10% | 0.60% | 0.50% |
| Glaucoma | 4.40% | 9.00% | 10.20% | 9.00% |
| Other_chronic_disease | 16.70% | 12.60% | 22.90% | 16.40% |
| other_illness | 13.90% | 12.00% | 10.20% | 12.60% |
